# High-Intensity Interval Training Decreases Circulating HMGB1 in Individuals with Insulin Resistance: Plasma Lipidomics Correlate with Associated Cardiometabolic Benefits

**DOI:** 10.31083/FBL31396

**Published:** 2025-03-21

**Authors:** Gabriela Martinez Bravo, Prabu Paramasivam, Gabriella F. Bellissimo, Quiteria Jacquez, Huayu Zheng, Fabiano Amorim, Lara Kovell, Roberto Ivan Mota Alvidrez

**Affiliations:** 1 Pharmaceutical Sciences, College of Pharmacy, University of New Mexico, Albuquerque, NM 87106, USA; 2 Biomedical Engineering Department, University of New Mexico, Albuquerque, NM 87131, USA; 3 Clinical and Translational Sciences Center, University of New Mexico, Albuquerque, NM 87131, USA; 4 Health, Exercise and Sports Sciences, University of New Mexico, Albuquerque, NM 87131, USA; 5 Cardiovascular Medicine, UMass Chan Medical School, Worcester, MA 01655, USA; 6 Cardiovascular and Metabolic Diseases (CVMD) Signature Program, University of New Mexico, Albuquerque, NM 87131, USA; 7 Autophagy, Inflammation, Metabolism CoBRE, University of New Mexico, Albuquerque, NM 87131, USA

**Keywords:** *HMGB1*, exercise, insulin resistance, HIIT, BW-HIIT, inflammation

## Abstract

**Background:**

Bodyweight high-intensity interval training (BW-HIIT) is an effective, time-efficient exercise method that reduces cardiovascular risk factors and improves muscle endurance without requiring external equipment. High mobility group box 1 (HMGB1) is a proinflammatory protein involved in insulin resistance. Previous studies revealed that *HMGB1* knockout mice show improved insulin sensitivity and hyperglycemia. This study investigates whether BW-HIIT exercise can reduce proinflammatory markers, such as HMGB1, in individuals with insulin resistance.

**Methods:**

In total, 14 adults (2 male/12 female) aged 18 to 55 were subject to six weeks of BW-HIIT. Additionally, 10-week-old mice were subject to exercise conditioning (5 mice per group (all male)) for 4 weeks of treadmill exercise or sedentary. Human and mouse pre- and post-exercise serum/plasma samples were analyzed for lipidomics, hormonal, and cytokine multiplex assays. Cardiometabolic parameters were also performed on human subjects.

**Results:**

Post-exercise decreased systolic blood pressure (SBP), cholesterol, triglycerides, high-density lipoprotein (HDL), and cholesterol/HDL ratio in human patients with insulin resistance. Meanwhile, hormones such as amylin, glucagon, and insulin all increased post-BW-HIIT or treadmill exercise in both human and mouse models. Moreover, circulating HMBG1 levels were reduced in insulin-resistant individuals and mice after exercise. Furthermore, treadmill exercise by the animal model increased anti-inflammatory cytokines, including interleukin (IL)-10, IL-12p40, and IL-12p70, and reduced proinflammatory cytokines: eotaxin, IL-2, and macrophage inflammatory protein (MIP)-2 or CXCL2.

**Conclusions:**

Six weeks of BW-HIIT exercise can improve cardiometabolic health, anti-inflammatory markers, hormones, and insulin sensitivity in human and mouse models undergoing exercise. Changes in circulating HMBG1 levels following BW-HIIT exercise make HMGB1 a suitable marker for cardiometabolic disease, potentiating its role beyond an alarmin. Further studies are needed to confirm these effects and to elucidate the underlying physiological mechanisms.

## 1. Introduction

Insulin resistance is a clinical condition where muscle, fat, and liver cells become less responsive to insulin, leading to impaired glucose uptake and elevated blood glucose levels [1]. Persistent hyperglycemia ensues, culminating in Type 2 Diabetes (T2D), when the pancreas no longer produces sufficient and effective insulin to manage blood glucose levels [2,3]. Early intervention through lifestyle changes such as improved diet and increased physical activity is crucial to prevent or delay the onset of T2D [3–5].

Exercise therapy is a cornerstone in managing various chronic diseases, including T2D and inflammatory conditions known to benefit glucose control [6]. According to a systematic review [7], exercise interventions lasting over eight weeks led to significant reductions in glycated hemoglobin A1C (HbA1c), fasting blood glucose levels, body mass index (BMI), and waist circumference. Exercise optimizes glycemic control and enhances the overall quality of life for individuals with T2D [7,8]. Regular physical activity, including high-intensity exercises like high-intensity Interval Training (HIIT) and moderate exercises like walking, is recommended to complement medical treatments for effective diabetes management [9]. HIIT involves short bursts of intense exercise followed by periods of rest or low-intensity exercise [10,11]. This training enhances insulin sensitivity through several possible mechanisms: (1) HIIT increases glucose uptake by muscle cells by enhancing the activity of glucose transporter Type-4 (GLUT4), which facilitates glucose entry into the cells. (2) It improves mitochondrial function and increases the number of mitochondria in muscle cells, which enhances their capacity to oxidize glucose and fatty acids for energy. (3) HIIT promotes fat loss, particularly visceral fat, and is strongly associated with insulin resistance. (4) HIIT reduces inflammation and oxidative stress, which can impair insulin signaling pathways. These physiological changes collectively improve insulin sensitivity and blood glucose control [10,12,13]. The review by Shah *et al*. (2021) [9] found that exercise interventions reduced HbA1c by 0.5% to 0.8% on average, indicating significant improvements in long-term glycemic control. A key factor in the regulation and progression of insulin resistance is the nuclear protein highmobility group box 1 (*HMGB1*). This active protein is particularly significant during oxidative stress and inflammation as it triggers various inflammatory signaling pathways, including the toll-like receptors (TLRs) and receptors for advanced glycation end products (RAGE) [14]. Activating these receptors produces pro-inflammatory cytokines [15], such as tumor necrosis factor (TNF)-*α* and interleukin (IL)-6, which disrupt insulin signaling pathways [14].

Our group’s research has yielded encouraging results in recent years on the role of *HMGB1* in insulin resistance and T2D. We have expanded our understanding of *HMGB1* beyond its role as an alarmin, identifying it as a promising therapeutic target in T2D progression [16]. In our recent mice studies, we have found that inhibiting *HMGB1* activity in mice improves insulin sensitivity and reduces hyperglycemia, and by RNA sequencing analyses, we identified potential alternations in genes and molecular pathways that may account for the *in vivo* phenotypic changes observed in hyperglycemic mice, suggesting *HMGB1*’s potential in addressing insulin resistance [16]. In this study, we investigate the impact of body weight (BW)-HIIT on circulating *HMGB1* levels as a potential intervention for insulin resistance. We hypothesize that exercise training lowers *HMGB1* levels, thereby reducing its pro-inflammatory effects and enhancing insulin sensitivity in individuals with insulin resistance.

## 2. Materials and Methods

### 2.1 Human Exercise Model

For this study, all individuals were informed of their enrolment objectives, procedures, potential risks, discomforts, and benefits. Informed consent was obtained before participation. The University of New Mexico (UNM) Institutional Review Board (IRB) Main Campus approved this study with reference number 21–3,19.

Fourteen individuals (2 male/12 female) were enrolled in the study, meeting the following inclusion criteria: (1) adults ages 18 to 55 years, (2) classified with obesity (BMI ≥30 kg/m^2^) but non-insulin resistant (diagnosed by Homeostatic Model Assessment for Insulin Resistance (HOMA-IR) index, which is described below), (3) physically inactive (<150 minutes of moderate to vigorous intensity physical activity per week), and (4) nonsmokers. The exclusion criteria were individuals who reported taking glucose-lowering or lipid-lowering medications; another exclusion criteria were patients with diagnosed prediabetes or T2D that determined by their clinical history, questionnaire, and physician diagnosis. We also took random measurements of their glucose to ensure the levels were <200 mg/dL, analyzed by a portable glucose meter (TRUE METRIX Blood Glucose Monitor^™^, Trividia Health, Inc., Ft. Lauderdale, FL, USA). Before starting the study, blood glucose levels were analyzed by sending samples to a commercial lab (Quest Direct^™^, Albuquerque, NM, USA) for fasting blood glucose and HbA1c, for which all values were reported below to be below 5.7%.

Baseline and post-testing procedures were performed at the UNM Exercise Physiology laboratory. Individuals were instructed to arrive in a hydrated and fasted state for ≥8 hours and to abstain from alcohol and vigorous exercise for 24 hours. Before body composition measurement, hydration status was checked using urine specific gravity (USG), and individuals with >1.020 were considered dehydrated and therefore excluded. Next, individuals were provided with a Fitbit Inspire 3 (Model: FB424; Fitbit, Inc., San Francisco, CA, USA) wearable activity tracking (WAT) device. This electronic monitoring device enables users to track health-related fitness metrics such as steps, activity level, walking distance, heart rate, and sleep patterns [17] and instructs them to install the Fitbit smartphone application (Fitbit, Inc., San Francisco, CA, USA). After the WAT was set up, individuals completed maximal voluntary isometric strength tests and treadmill peak oxygen consumption (VO_2_peak) tests. Post-testing data collection procedures were the same as the baseline testing apart from the glucometer check and setting up the individual’s WAT device. All excluded individuals were provided with their results and advised to seek further medical screening. Blood samples (~15 mL) were collected from individuals fasting from the antecubital vein. Samples were collected in anti-coagulant treated EDTA tubes for plasma separation or untreated tubes to allow the blood to clot by leaving it undisturbed at room temperature. Serum was obtained by centrifuging the tubes for 15 min (1000 g, 22 °C) (Allegra X-14R Centrifuge, Beckman Coulter, Brea, CA, USA) and stored at −80 °C for subsequent analysis. Pre- and post-intervention quantitative measurements of leptin (Crystal Chem High-Performance Assays, Inc., Chicago, IL, USA) and IL-6 (Quantikine R&D System, Minneapolis, MN, USA) were assessed using enzyme-linked immunosorbent assays (ELISA) kits for human *HMGB1* (MBS701378, MyBioSource, San Diego, CA, USA) and mouse *HMGB1* (#MBS722248, MyBioSource, San Diego, CA, USA). ELISA kit procedures were performed according to the manufacturer’s instructions, and the average intraassay coefficient of variations was 2.9% for leptin and 24% for IL-6. Serum concentrations of insulin, glucose, low-density lipoprotein (LDL), high-density lipoprotein (HDL), triglycerides (TG), total cholesterol (TC), and hs-CRP, and the EDTA sample for HbA1c were sent to a commercial laboratory (Quest Direct^™^, Albuquerque, NM, USA). Insulin resistance was calculated using HOMA-IR (fasting insulin (uIU/mL) × fasting glucose (mg/dL)/405) [18,19].

After all baseline procedures, individuals were instructed on how to perform the remote exercise program. The program involved maximal effort BW-HIIT, prescribed three days a week over 6 weeks; individuals were allowed to exercise on days that best suited their schedule [20]. The BW-HIIT workouts were YouTube-based, consisting of a two-minute warm-up, two sets of five intervals, and a two-minute rest period after the first five intervals, for 27 minutes total. The progression of the videos was every two weeks, involving an increase in the duration of the maximal effort work interval and a decrease in the duration of the recovery interval (i.e., week 1–2: 30 s work × 90 s recovery, week 3–4: 40 s work × 80 s recovery, week 5–6: 60 s work × 60 s recovery). The YouTube BW-HIIT-based videos are available at: https://youtu.be/EbeTy72VtuM?si=9IXF Dnuhd7–5-nLI. During the 6 weeks of remote training, individuals were provided an exercise journal to record their overall rating of perceived exertion (RPE) immediately after each workout. Additionally, they used their wearable activity tracker (WAT) to log heart rate after each maximal effort interval. The WAT also monitored program compliance by tracking physical activity (PA) minutes recorded on participants’ Fitbit profiles [21]. After 6 weeks, the individuals returned to the lab, where we performed the same analysis as the baseline pre-exercise visit. The evaluation of the changes in cardiometabolic markers were evidenced by the percentage of change from baseline (pre-exercise) to post-exercise in inflammatory markers (CRP and IL-6), glycemic markers (HbA1c), and Leptin (marker useful in feeling full and satiety) to investigate the effects of exercise. We performed our analysis comparing the changes in non-insulin resistant vs. insulin resistant individuals. From our study individuals we determined insulin resistant was via HOMA-IR calculation. Individuals with HOMA-IR <2 were considered non-insulin resistant. In contrast, those with HOMA-IR >2 were considered insulin resistant.

### 2.2 Animal Model of Exercise Training

All animal experiments were approved by the ethics committee of the University of New Mexico (UNM) Institutional Animal Care and Use Committee (IACUC) and Animal Welfare Committee. All live animal studies were conducted ethically, following relevant guidelines and regulations at UNM under protocol number 23–201405-HSC. All animals were purchased from The Jackson Laboratory (Bar Harbor, ME, USA), bred and housed under the Animal Resource Facilities (ARF) at UNM. Adult male mice 5 per group (based on previous studies power analysis showing power of 80% for 15% differences in measurements of glucose controls with an N of 5 per group) were randomized and assigned in unbiased conditions at 10 weeks of age under 2 groups: (1) sedentary controls, no exercise, and (2) the exercised mice, who ran on treadmills at 80 meters/min for 1 hr/day, 5 days/week for 4 weeks. Both groups were on a normal diet. All exercise mice were acclimated to treadmills for 1 week before starting the study, to ensure they were comfortable running at the set speed and duration planned. We collected blood at 12 weeks old for baseline and at 16 weeks old at the end of the exercise period (and same for the age-matched sedentary mice). At the end of the experimental protocol, the animals were euthanized using a dose of 0.01 mL/g of Ketamine/Xylazine. Whole blood was collected for serum and plasma isolation. The aorta, liver, and muscle tissues were harvested. For Insulin resistant mouse development, we implemented a high-fat diet (HFD) (D12492, Research Diets) that was administrated starting at 6 weeks of age before exercise training. For T2D model development, an HFD (D12492, Research Diets) was administrated at 6 weeks of age [22]. At 8 weeks of age, an intraperitoneal (IP) injection of streptozotocin (STZ) was given for 5 consecutive days at a dosage of 25 mg/kg to the mice because this induces hyperglycemia and insulin resistance. Following the final STZ injection, the mouse model was monitored and allowed to evolve over 10 weeks. These mice were placed in treadmills to run daily for 1 hr at a velocity of 80 meters/min for 4 weeks [16,23–25]. We used a non-exercise *HMGB1* Flox mice that were fed HFD (D12492, Research Diets) for 6 weeks as previously performed [16]. Briefly, inducible *HMGB1* knockdown was achieved using Tamoxifen (TMX) injections administered to the mice at 6 weeks of age via IP injection at 1 mg/kg over 10 days. For induction of diabetic phenotype in mice, the same TMX procedure was followed as described above but after a 7-day post-TMX injection period, mice were administered a low dose of STZ via IP for 5 consecutive days at a dosage of 25 mg/kg. Diabetic condition was monitored for 10 weeks. An ELISA kit (MyBioSource, MBS701378) was used to measure the levels of circulating *HMGB1* in all our mice.

### 2.3 Human and Murine Cytokine and Hormonal Blood Panel Analysis

Human pre- and post-exercise serum were sent to Eve Technologies Corporation (Calgary, Alberta, Canada) to perform the Human Hormone 12-Plex Discovery Assay (MilliporeSigma, Burlington, MA, USA). This assay uses Luminex xMAP technology for multiplexed quantification of 12 Human cytokines, chemokines, and growth factors. The multiplexing analysis was performed using the Luminex^™^ 200 system (Luminex, Austin, TX, USA) by Eve Technologies Corp.. According to the manufacturer’s protocol, twelve markers were simultaneously measured in the samples using Eve Technologies’ Human Hormone 12-Plex Discovery Assay^®^. The 12-plex consisted of Amylin (active), C-Peptide, Ghrelin, Gastric inhibitory polypeptide (GIP), Glucagon-like peptide-1 (GLP-1) (active), Glucagon, Insulin, Leptin, Monocyte chemoattractant protein-1 (MCP-1), Pancreatic polypeptide (PP), Peptide tyrosine tyrosine (PYY), and Secretin. Assay sensitivities of these markers range from 0.6–46.9 pg/mL for the 12-plex. Individual analyte sensitivity values are available in the MilliporeSigma MILLIPLEX^®^MAP protocol as followed in the link provided below for the specific manufacturer (https://www.sigmaaldrich.com/US/en/configurators/milliplex?srsltid=AfmBOopvpI4zLdIIASdczaOdy6oAQB0jEN1xq6GFT2BTgx1BOIPxr2n9).

For the human Cytokine Panel 4 12-Plex Discovery Assay (MilliporeSigma, Burlington, MA, USA), Luminex xMAP technology was used for multiplexed quantification of 12 human cytokines, chemokines, and growth factors. The multiplexing analysis was performed using the Luminex^™^ 200 system (Luminex, Austin, TX, USA) by Eve Technologies Corp. (Calgary, Alberta). According to the manufacturer’s protocol, twelve markers were simultaneously measured in the samples using Eve Technologies’ Human Cytokine Panel 4 12-Plex Discovery Assay^®^. The 12-plex consisted of B-cell activating factor (BAFF), Breast and kidney-expressed chemokine (BRAK), C-X-C motif chemokine ligand 16 (CXCL16), C-C motif chemokine ligand 16 (HCC-4), *HMGB1*, Interferon beta (IFN*β*), Interleukin-24 (IL-24), Interleukin-28B (IL-28B), Interleukin-35 (IL-35), Interleukin-37 (IL-37), Macrophage inflammatory protein-4 (MIP-4), and Chitinase 3-like protein 1 (CHI3L1). Assay sensitivities of these markers range from 2.0–3800 pg/mL for the 12-plex. Individual analyte sensitivity values are available in the MilliporeSigma MILLIPLEX^®^ MAP protocol.

For mice, serum from the 2 groups was isolated and sent to Eve Technologies Corporation to perform the Mouse Hormone 12-Plex Discovery Assay (Millipore-Sigma, Burlington, MA, USA); this study used Luminex xMAP technology for multiplexed quantification of 12 Mouse cytokines, chemokines, and growth factors. The multiplexing analysis was performed using the Luminex^™^ 200 system (Luminex, Austin, TX, USA) by Eve Technologies Corp. (Calgary, Alberta). According to the manufacturer’s protocol, twelve markers were simultaneously measured in the samples using Eve Technologies’ Mouse Hormone 12-Plex Discovery Assay^®^. The 12-plex consisted of Amylin (active), C-Peptide 2, Ghrelin, GIP (total), GLP-1 (active), Glucagon, Insulin, Leptin, PP, PYY, Resistin and Secretin. Assay sensitivities of these markers range from 1.4–91.8 pg/mL for the 12-plex. Individual analyte sensitivity values are available in the MilliporeSigma MILLIPLEX^®^ MAP protocol.

Mouse Cytokine 32-Plex Discovery Assay (Millipore-Sigma, Burlington, MA, USA) was performed using Luminex xMAP technology for multiplexed quantifying 32 Mouse cytokines, chemokines, and growth factors. The multiplexing analysis was performed using the Luminex^™^ 200 system (Luminex, Austin, TX, USA) by Eve Technologies Corp. (Calgary, Alberta). According to the manufacturer’s protocol, thirty-two markers were simultaneously measured in the samples using Eve Technologies’ Mouse Cytokine 32-Plex Discovery Assay^®^. The 32-plex consisted of Eotaxin, Granulocyte colony-stimulating factor (G-CSF), Granulocyte-macrophage colony-stimulating factor (GM-CSF), Interferon-gamma (IFN*γ*), Interleukin-1 alpha and beta (IL-1*α*, IL-1*β*), Interleukins (IL-2, IL-3, IL-4, IL-5, IL-6, IL-7, IL-9, IL-10, IL-12(p40), IL-12(p70), IL-13, IL-15, IL-17), C-X-C motif chemokine 10 (CXCL10) (IP-10), Keratinocyte-derived cytokine (KC), Leukemia inhibitory factor (LIF), Epithelial-derived neutrophil-activating peptide 78 (LIX), Monocyte chemoattractant protein-1 (MCP-1), Macrophage colonystimulating factor (M-CSF), Monokine induced by gamma interferon (MIG), Macrophage inflammatory protein (MIP-1*α*, MIP-1*β*, MIP-2), Regulated on activation, normal T cell expressed and secreted (RANTES), tumor necrosis factor (TNF-*α*), and Vascular endothelial growth factor (VEGF). Assay sensitivities of these markers range from 0.3–30.6 pg/mL for the 32-plex. Individual analyte sensitivity values are available in the MilliporeSigma MILLIPLEX^®^ MAP protocol.

### 2.4 Plasma Metabolomic/Lipidomic Analysis

Untargeted metabolomic analysis of plasma samples was conducted using MxP^®^ Quant 500 kit (Biocrates, Innsbruck, Austria), in which 630 metabolites from 26 biochemical classes were assessed. Sample preparation, data acquisition method, and data processing followed the manufacturer’s protocols. In brief, 10 μL of blank solution (Phosphate buffered saline), calibration standard solutions, quality control solutions, and human plasma samples were added into a 96-well plate pre-incorporated with internal standards provided by Biocrates. The plate was dried under nitrogen flow using a positive pressure manifold (Positive Pressure-96 Processor, Waters Corporation, Milford, MA, USA). Then, a derivatization solution containing 5% phenyl isothiocyanate was added to all the wells, and the plate was incubated for 60 min at room temperature (RT) followed by another drying step with the manifold. An extract solvent of 5 mM ammonium acetate in methanol was added to each well, followed by shaking at RT for 30 min. The extracts were eluted into a new 96-well plate using the manifold and further diluted with either ultrapure water or flow injection analysis (FIA) solvent (provided with the Biocrates kit) for liquid chromatographytandem mass spectrometry (LC-MS/MS) and FIA tandem mass spectrometry (FIA-MS/MS), respectively. The LC-MS/MS and FIA-MS/MS measurements were performed to quantify small molecules and lipids, respectively, using an ExionLCTM UHPLC system coupled to a 5500 QTRAP^®^ a triple quadrupole mass spectrometer (AB Sciex, Darmstadt, Germany). The LC-MS/MS at positive and negative ion modes was run first. The LC column was an MxP^®^ Quant 500 kit system column system (provided with the Biocrates kit). Mobile phase compositions, temperature, and LC gradient elution conditions were set following Biocrates’ methods. The FIA-MS/MS test was run after the column had been removed. The data were acquired using the Analyst (Sciex) software (https://sciex.com/products/software/analyst-software) with multiple reaction monitoring (MRM) for all metabolites with optimized MS parameters provided by the manufacturer. WebIDQ cloud software (Biocrates, Innsbruck, Austria) was used for data processing to validate, quantify, and export data. The concentrations (μM) for each metabolite in different classes were calculated using WebIDQ. MetaboINDICATOR provided the relevant pathways and biological functions linked with metabolite sums and ratios as a built-in tool from WebIDQ.

### 2.5 Statistical Analysis

Insulin resistance was calculated using HOMA-IR (fasting insulin (uIU/mL) × fasting glucose (mg/dL)/405) [26], and insulin resistant sub-analysis was performed regardless of sex but based on HOMA-IR. A sub-analysis was evaluated, and then pre- and post-exercise measurements were taken. For cardiometabolic and metabolomic analysis, statistical analyses were performed using IBM SPSS (IBM Corp., Version 23.0, Armonk, NY, USA). All illustrated data was performed using GraphPad Prism v8.4 (GraphPad Software, Inc., San Diego, CA, USA). Unpaired two-tailed Student’s *t*-tests and one-way ANOVA were performed when appropriate and detailed in each section and each figure legend. Figure legends specify the number of replicates and samples analyzed. *p*-values < 0.05 were determined to be significant. Unless otherwise indicated, data are presented as mean ± standard error (SE). The specific number of subjects is described for each assay. Statistical significance was set at a *p* value of *: <0.05, **: 0.001–0.01, ***: 0.0001–0.001, and ****: <0.0001.

## 3. Results

### 3.1 Inflammatory and Hormonal Panels Evidence Distinct Responses to Exercise in Humans and Mice

Circulating cytokine and chemokines post-exercise evidence heterogeneous effects in analysis between humans and mice exercise training. Plasma multiplex analysis demonstrated longitudinal changes in circulating levels of cytokines, chemokines, and growth factors in mice and humans pre- and post-exercise (Fig. 1A, mice). Anti-inflammatory cytokines, particularly IL-10 (*p*: 0.0471), IL-12p40 (*p*: 0.0443), IL-12p70 (*p*: 0.0009) and MIP-2 (*p* = 0.0039) were significantly increased in exercise-conditioned mice. Contrastingly, human cytokine and chemokines were not significantly modified post-exercise (Fig. 1B, human).

Plasma hormonal analysis revealed significant heterogeneity in the groups of hormone responses to exercise conditioning. Both mice and humans showed an increase in several hormones, indicating the positive impact of exercise in the gut, adipokine, and insulin levels in BW-HIIT and exercise animals. Our findings had high variability between groups (Fig. 2). In humans, the hormone with a bigger increase post-exercise was amylin, with a change from 35.64 pg/mL to 50.0 pg/mL (Fig. 2A). C-peptide increased from 2685.77 pg/mL to 2948.26 pg/mL (Fig. 2B). Glucagon increased from 36.65 pg/mL to 41.53 pg/mL, (Fig. 2C). Glucagon Inhibitory Peptide-1 increased from 24.63 pg/mL to 30.915 pg/mL (Fig. 2H). Contrastingly, some hormones showed more heterogeneity between mice and humans. Pancreatic Peptide (Fig. 2D), Ghrelin (Fig. 2E) and Insulin post-exercise was significantly higher in humans but showed no meaningful change in mice (Fig. 2F). In the other hand, mice showed an increase in amylin from 53.76 pg/mL to 59.66 pg/mL (Fig. 2A). In C-peptide the average increased from 157.61 pg/mL to 227.99 pg/mL (Fig. 2B). Glucagon increased from 22.49 pg/mL to 45.81 pg/mL (Fig. 2C). Glucagon Inhibitory Peptide-1 increased from 30.79 pg/mL to 64.92 pg/mL (Fig. 2H). Peptide YY (Fig. 2G), and Leptin evidenced a considerable increase in mice, but no substantial change in humans (Fig. 2I). Unmatched comparisons of Secretin levels increased post-exercise in mice from 13.00 pg/mL to 23.82 pg/mL (Supplementary Fig. 1A) as well as in Resistin (Supplementary Fig. 1B).

### 3.2 Reduced Cardiometabolic Risk Factor in Insulin Resistant Individuals Post-Exercise

As mentioned before the segregation of our population was given by the % of change in the HOMA-IR calculation. Our results show reduced cardiometabolic risk factors post-exercise in individuals with insulin resistance compared to non-insulin resistant individuals. Cardiometabolic risk factors such as systolic blood pressure decreased −4.2% (Fig. 3D), cholesterol −2.8% (Fig. 3F), non-HDL −1.4% (Fig. 3I) and cholesterol/HDL ratio −0.7% (Fig. 3H) in insulin resistant individuals compared to non-insulin resistant individuals. At the same time, pro-inflammatory markers like C-reactive protein decreased by 6.60% (Fig. 3A) and IL6 by 0.02% (Fig. 3B) in insulin-resistant participants individuals. Adipokine marker Leptin was decreased −7.4% (Fig. 3C) in insulin resistant individuals than in non-insulin resistant individuals. On the other hand, only a 0.6% decrease in HbA1c change in insulin resistant individuals compared to non-insulin resistant controls (Fig. 3E). These findings underscore the efficacy of home-based BW-HIIT in improving cardiometabolic health, particularly in individuals with insulin resistance. There was no significant change in Triglyceride levels between groups (Fig. 3G).

### 3.3 Plasma Metabolomic and Lipidomic Analysis Reveals a Beneficial Decrease in Circulating Lipid Mediators Post Exercise in Insulin Resistant Individuals

Lipidomic analysis post exercise was performed in non-insulin resistant and insulin-resistant individuals. We aim to illustrate the differential lipidomic profiles between each group in Fig. 4. Heat Map Analysis revealed significant clustering of lipidomic changes between pre- and post-exercise samples in both groups. Insulin resistant individuals exhibited a distinct decreased lipidomic profile compared to their non-insulin resistant counterparts, indicating a more pronounced response to the exercise regimen (Fig. 4A). Empirical bayesian analysis of microarray (EBAM) analysis identified several lipid species with a significant decrease from pre- to post-exercise almost uniquely in insulin resistant individuals (Fig. 4B). Principal component analysis (PCA) loading plot demonstrated a clear separation between pre- and post-exercise lipid profiles. The post-exercise lipidomic profiles of insulin resistant individuals clustered distinctly from their pre-exercise profiles and the profiles of non-insulin resistant individuals, suggesting specific lipidomic shifts attributable to a decrease in HOMA-IR with insulin resistance and exercise. (Fig. 4C). Self-organizing maps (SOM) plot highlighted greater variability in the lipidomic data of insulin resistant individuals compared to non-insulin resistant. Such variability exemplified the heterogeneity in cardiometabolic responses to BW-HIIT exercise within the insulin resistant group (Fig. 4D). Finally, both supervised partial least squares (SPLS) and orthogonal partial least squares (OPLS) plots, confirmed the lipidomic differences between pre- and post-exercise states. These analyses provided robust models for predicting polarized beneficial lipidomic changes based on exercise intervention, particularly in insulin resistant individuals (Fig. 4E,F). The fold change (FC) plot showed significant upregulation and downregulation of previously demonstrated beneficial lipids in insulin resistant individual’s post-exercise (Fig. 4G). The linear correlation heat map revealed a stronger correlation in insulin resistant individuals post-exercise to reduced harmful circulating lipid compared to non-insulin resistant individuals. This suggests that the effects of modulation of insulin resistance may allow for the enhancement of exercise driven benefits to metabolomic and lipidomic response (Fig. 4H).

### 3.4 HMGB1 and HOMA-IR Levels Decrease Post-exercise in Insulin Resistant Individuals, Effect is Mimicked in Mice

We then aimed to show the impact of BW-HIIT of exercise in HOMA-IR and *HMGB1* levels. We performed paired and segmented comparisons, delta changes, and comparisons with non-T2D and sedentary chronic T2D individuals (Fig. 5). A side-by-side clustered and segmented comparison confirms that insulin resistant individuals showed a wider distribution of HOMA-IR than non-insulin resistant control subjects (Fig. 5A). Paired comparison of HOMA-IR levels pre- and post-exercise shows a significant decrease in insulin resistant individuals, while non-insulin resistant individuals exhibit less change. This indicates that the improvement of insulin sensitivity is more pronounced in the insulin resistant group post-exercise (Fig. 5B). Perceived change from baseline (Delta, presented as the change from baseline) shows decreased HMBG1 levels post-exercise compared to pre-exercise individuals involved in the BW-HIIT exercise program (Fig. 5C). Sub-group analysis shows HMBG1 delta changes are augmented post-exercise (Fig. 5D). A sub-analysis identified increased serum HMBG1 levels in sedentary chronic T2D individuals compared to baseline and 6 weeks after exercise of non-T2D (Fig. 5E). Mice comparison correlates human findings shown in Supplementary Fig. 2A–C. *HMGB1* levels show similar results as humans, having a significant decrease in the post-exercise group. These results suggest that BW-HIIT exercise significantly reduces HOMA-IR and *HMGB1* levels in insulin resistant individuals, reflecting possible improvement in insulin sensitivity and reduced inflammation. The consistent findings across human and mouse models highlight the potential of BW-HIIT exercise as an effective intervention for cardiometabolic health improvement in insulin resistance with a strong concept of T2D, paving the way for a brighter future in cardiometabolic health research.

## 4. Discussion

Our study gives novel evidence, to our knowledge, of the effect of decreased circulating *HMGB1* in insulin resistant individuals performing maximal effort BW-HIIT due to its cardiometabolic benefits. In addition, we compared these results with mouse model following continuous treadmill running seeking to correlate between murine and human results. Importantly, we found vast heterogeneity between mouse and human exercise conditioning. We showed that even remote training has a high impact on insulin secretion and sensitivity after exercise but mostly observed in human studies, even though pancreatic peptides are enhanced with exercise in both models. Plasma lipidomics demonstrated the significant advantages of BW-HIIT in reducing harmful circulating lipids in insulin resistant individuals. We conducted this study in individuals using the modified BW-HIIT model to highlight the potential cardiometabolic benefits, especially for individuals unable to perform conventional aerobic exercise training regimens using specialized exercise equipment. We also created a continuous exercise treadmill model for mice with insulin resistance, focusing on comparing findings in circulating *HMGB1* and proinflammatory mediators. Our findings show: (i) the anti-inflammatory cytokines IL-10, IL-12p40, and IL-12p70 increased with pro-inflammatory cytokines Eotaxin, and MIP-2 or CXCL2 increased in post-exercise mice; (ii) the hormones like Amylin, Glucagon, and Insulin increased in post-exercise human and mouse models; (iii) post-exercise, insulin resistant individuals show a higher decrease in cardiometabolic risk factors including systolic blood pressure, cholesterol, triglycerides, HDL, and Chol/HDL ratio with only 6 weeks of remote training; (iv) similar to decreases in HOMA-IR, BW-HIIT reduced circulating HMBG1, particularly in insulin resistant individuals. BW-HIIT has distinct effects on hormones across the two groups due to baseline characteristics, training protocols, and physiological adaptability variations. The observed hormonal changes include decreased leptin levels due to decreased fat mass and improved leptin sensitivity, enhanced insulin sensitivity by reducing inflammation, improved lipid metabolism, and augmented muscle glucose uptake [27]. BW-HIIT reduces insulin levels immediately after exercise by enhancing glucose uptake through non-insulin-dependent pathways. As mentioned before, its effects on *HMGB1* levels and insulin sensitivity are mediated through multiple pathways, including reductions in oxidative stress and inflammation and modulation of *HMGB1* expression and release [28]. BW-HIIT enhances mitochondrial function and biogenesis, increasing the efficiency of energy production and reducing the generation of reactive oxygen species (ROS). This oxidative stress induces *HMGB1* acetylation, promoting its release from the nucleus to the extracellular environment. By reducing ROS, BW-HIIT inhibits this release, lowering extracellular *HMGB1* levels [29].

Recent studies suggest that aerobic exercise increases pro-oxidant status more than anaerobic exercise, and aerobic and anaerobic exercise activities improve antioxidants [30,31]. At the same time, it has been demonstrated that increasing exercise intensity resulted in more excellent endogenous antioxidant defenses [32–35]. HIIT, a form of exercise that is primarily anaerobic, is characterized by short bursts of intense activity followed by rest periods [36,37], consequently relying heavily on anaerobic energy pathways [38]. This style of training is a well-known stimulus enhancing anaerobic capacity and aerobic fitness [39]. Our research findings demonstrate that cardiometabolic risk factors, a cluster of factors that increase the likelihood of developing cardiovascular disease and metabolic disorders such as T2D, can be effectively reduced through regular exercise [40,41]. HIIT’s benefits improve endothelial function, reduce inflammation, and enhance vascular health, thereby lowering blood pressure, improving lipid profiles, and reducing excess body weight [42–45]. Our study on insulin resistance individuals who underwent BW-HIIT demonstrated reduced cardiometabolic risk factors such as systolic blood pressure and cholesterol. Similarly, recent studies have shown positive changes in cardiometabolic risk factors, including systolic blood pressure, in obese individuals undergoing HIIT or a mobile health diabetes prevention trial [46,47]. In contrast, Bouchonville *et al*. [48] reported that weight-loss-based lifestyle interventions, not exercise alone, effectively reduced multiple cardiometabolic risk factors in obese older adults. Our study implies that BW-HIIT offers a credible advantage and alternative to traditional styles of exercise training in improving cardiometabolic health and reducing hypertension in obese adults. Our research is directly relevant to exercise physiology benefits in diabetics. As such, amylin and insulin, co-secreted within pancreatic beta cells, are crucial in maintaining blood glucose levels [8,49]. We observed increased amylin and insulin levels in humans and mice post-exercise, suggesting that BW-HIIT post-exercise directly enhances insulin sensitivity. Conversely, glucagon, a key player in hepatic glycogenolysis and gluconeogenesis during exercise, was elevated in plasma in post-exercise humans and mice. Ghrelin, a regulator of appetite, energy balance, glucose metabolism, and insulin sensitivity, was also affected. Our study revealed post-exercise improves hormones and increases ghrelin levels in insulin-resistant individuals. These findings parallel those of a recent study that showed the effect of lifestyle intervention in individuals with obesity and insulin resistance, which showed a 23.6% reduction in Ghrelin levels after four months of intervention [50].

Our study demonstrated that HMBG1 levels can be reduced post-exercise in insulin resistant adults. Because as we mentioned before *HMGB1* plays a critical role in insulin resistance through its ability to act as a damage-associated molecular pattern (DAMP). Its interaction with receptors like toll-like receptors (TLRs) and the receptor for advanced glycation end products (RAGE) triggers pro-inflammatory and stress-related pathways, which impair insulin signaling [38]. A contributing factor is the ROS generated during oxidative stress, which damages cellular components and hampers insulin receptor function, thereby worsening insulin resistance [14,51]. This finding is significant and holds immense potential as it provides a potential mechanism for the beneficial effects of BW-HIIT exercise on insulin resistance. HMBG1, an essential DAMP-released protein, triggers inflammation that can infiltrate into tissue, a process strongly associated with insulin resistance [38,52,53]. Importantly, our findings align with those of Gmiat *et al*. [54], who observed decreased HMBG1 levels after 12 weeks of Nordic walking with vitamin supplementation by older women, suggesting that regular exercise may diminish the response in non-insulin resistant adults [55]. However, in contrast, the *HMBG1*, the receptor for advanced glycation end products and nucleosomes, increases after a marathon [56,57]. These results underscore the high potential but distinct acute vs. chronic effect of exercise training that can modulate circulating *HMGB1*, thereby serving as a valuable biomarker in both non-insulin resistant and clinical conditions. This potential opens exciting avenues for further research and clinical applications, especially for our group [58]. These findings also contrast acute vs chronic responses’ effect on *HMGB1* [59]. These findings, which echo those of a recent study on *HMBG1* levels pre and post marathon, suggest potential beneficial effects of marathon running on the body. The *HMGB1* level was analyzed 10–12 weeks prior, 1–2 weeks before, immediately, 24 h, 72 h, and 12 weeks post-race. The results demonstrated a significant increase in *HMGB1* from pre- to immediate post-race, followed by a return to baseline within 24–72 h. It is worth noting that strenuous exercise induces distinct changes in circulating *HMGB1*, and marathon running can trigger extensive responses from the body, potentially leading to beneficial effects, as observed in these subjects [51,52]. These findings direct us to future studies investigating recruiting and retaining more chronic T2D individuals and providing them with tailor-made supervised BW-HIIT exercise training that could improve their glycemic control, thereby retaining nuclear *HMGB1* signals. These implications for future research highlight the need for further investigation into the acute and chronic effects of exercise on *HMGB1* levels and the potential benefits of exercise on insulin resistance. As we discussed, it has become a significant biomarker in understanding glucose metabolism and the inflammatory processes associated with T2D and other medical conditions due to its involvement in inflammation and tissue damage [60]. Traditional biomarkers for T2D focus on blood glucose levels, HbA1c, and insulin sensitivity [61]. *HMGB1* is unique in its potential as a biomarker for diagnosing inflammatory conditions, assessing disease severity, and predicting patient outcomes [60]. Additionally, the acetylated form of HMGB1 (acetyl-*HMGB1*) is a modified form actively secreted by cells in response to stress and inflammation. This form is particularly potent in signaling and modulating immune responses, further influencing glucose metabolism. It has gained attention for its distinct functions and potential as a biomarker, further expanding medicine’s diagnostic and prognostic capabilities [51]. In conclusion, *HMGB1* and its variants are promising for improving disease management and advancing personalized medicine, emphasizing the importance of glucose control and inflammation in biomarker research.

We are mindful of our study’s limitations. BW-HIIT workouts are held three days a week over six weeks, and all study subjects were instructed remotely. This remote instruction could have led to variations in the intensity and adherence to the training program [20]. In this study, we establish a correlation between *HMGB1* and cardiometabolic benefits in insulin resistant individuals. Therefore, even though we used a small sample size (14 individuals), we accomplished a great goal of establishing a pivotal role for exercise training, even remotely, in decreasing insulin resistance and its correlation to cardiometabolic benefits and decreasing a marker of both glucose homeostasis and chronic inflammation. Our future studies will study bigger effects with the potential following these findings in Type-2 Diabetic patients since a small sample size might lead to wide confidence intervals, making effect size estimates (changes in hormones or insulin sensitivity) less precise. However, due to the limitation of cohort individuals, especially among the groups of only four non-insulin-resistant patients and ten insulin-resistant patients, this study reveals the main effect of systemic HMBG1 in insulin resistance on pre vs. post exercise changes in HMBG1 levels in preand post-exercise. The other strength is that these studies were conducted during COVID-19, as the patients had to be remote and had limitations to performing outdoor activities. Analyzing HMBG1 changes in pre- and post-exercise training is also challenging since some of our individuals showed variability in the extent of the exercise they performed [62]. This study aimed to evaluate *HMGB1* as a helpful biomarker, which will be ideal for assessing antihyperglycemic treatment efficacy in future studies [63]. Additionally, the study’s smaller size limits the generalizability of the findings. For this study, we focused firmly on retaining a group of individuals with insulin resistance since it is a population that struggles to find accurate exercise regimens. In the future, prospective studies will aim to recruit a higher number of subjects that are sex and agematched within non-insulin-resistant and T2D individuals with the presence and absence of diabetes-associated complications and provide them with tailor-made supervised BW-HIIT exercises. Furthermore, the study did not include a control sedentary group, which could have provided a better comparison of the effects of *HMGB1* and BW-HIIT exercise. However, a pilot study by Goh and Behringer [55] examined the impact of concurrent high-intensity aerobic and resistance exercise on releasing alarmins such as HMBG1 and inflammatory biomarkers in non-insulin resistant young men and found elevated plasma *HMBG1* levels immediately (30 min) after exercise each week and 24 h after the final exercise session in week 3 (24 h). Importantly, we cannot definitively exclude sex differences in the effects of exercise training on the *HMBG1* level. The last study limitation is the lack of large statistical differences caused by age and gender, which may influence the interpretation of *HMGB1*’s sensitivity and specificity as an adipokine indicator. Age is a critical factor influencing *HMGB1* levels, given its role in age-related inflammation, metabolic dysfunction, and oxidative stress. Older adults typically exhibit distinct adipokine profiles compared to younger individuals. This may alter the sensitivity and specificity of it. Future research should stratify participants by age or include age as a covariate in statistical models to validate the generalizability of *HMGB1* as an indicator across different age groups. Although the study presents valuable insights, the lack of consideration for age as a confounding variable underscores the need for a cautious interpretation of the findings [64]. These limitations should be considered when interpreting the study’s results.

As part of our future directions, we plan to extend the study, including a more significant population to address our limitations and age-matched chronic T2D patients beyond six weeks of training using supervised in-person training. We aim for future studies to measure *HMGB1* levels in blood and blood-derived extracellular vesicles to understand how *HMGB1* is released in insulin-resistant patients and those diagnosed with T2D and evidence how inhibiting the expression of *HMGB1* will improve body validation and give a cardioprotective effect. This next step is crucial and holds promise as it will help us understand the potential of extracellular HMBG1 in regulating insulin secretion in response to exercise training. These future studies will be able to determine the mechanistic role of HMBG1 in our understanding of diabetes management and obesity treatment. Furthermore, we aim to perform additional research to develop culturally adaptable versions of vigorous-intensity interval training using one’s body weight. Such studies will promote inclusive training protocols for diverse genders, demographics, and minorities in New Mexico and the US population and improve their quality of life.

## 5. Conclusions

The current study demonstrated that 6-weeks of bodyweight high intensity interval training improves cardiometabolic health, anti-inflammatory hormones, and insulin sensitivity in human and preclinical exercise mouse models. Correlatively, changes in circulating HMBG1 using BW-HIIT exercise training make *HMGB1* a suitable marker for cardiometabolic disease, potentiating its role beyond an alarmin. Further studies are needed to confirm these effects and to elucidate the underlying physiological mechanisms. Overall, these experiments reinforce the potential of BW-HIIT exercise interventions in insulin resistant adults to prevent the development of T2D.

## Supplementary Material

Supplemental material

Supplementary material associated with this article can be found, in the online version, at https://doi.org/10.31083/FBL31396.

## Figures and Tables

**Fig. 1.**
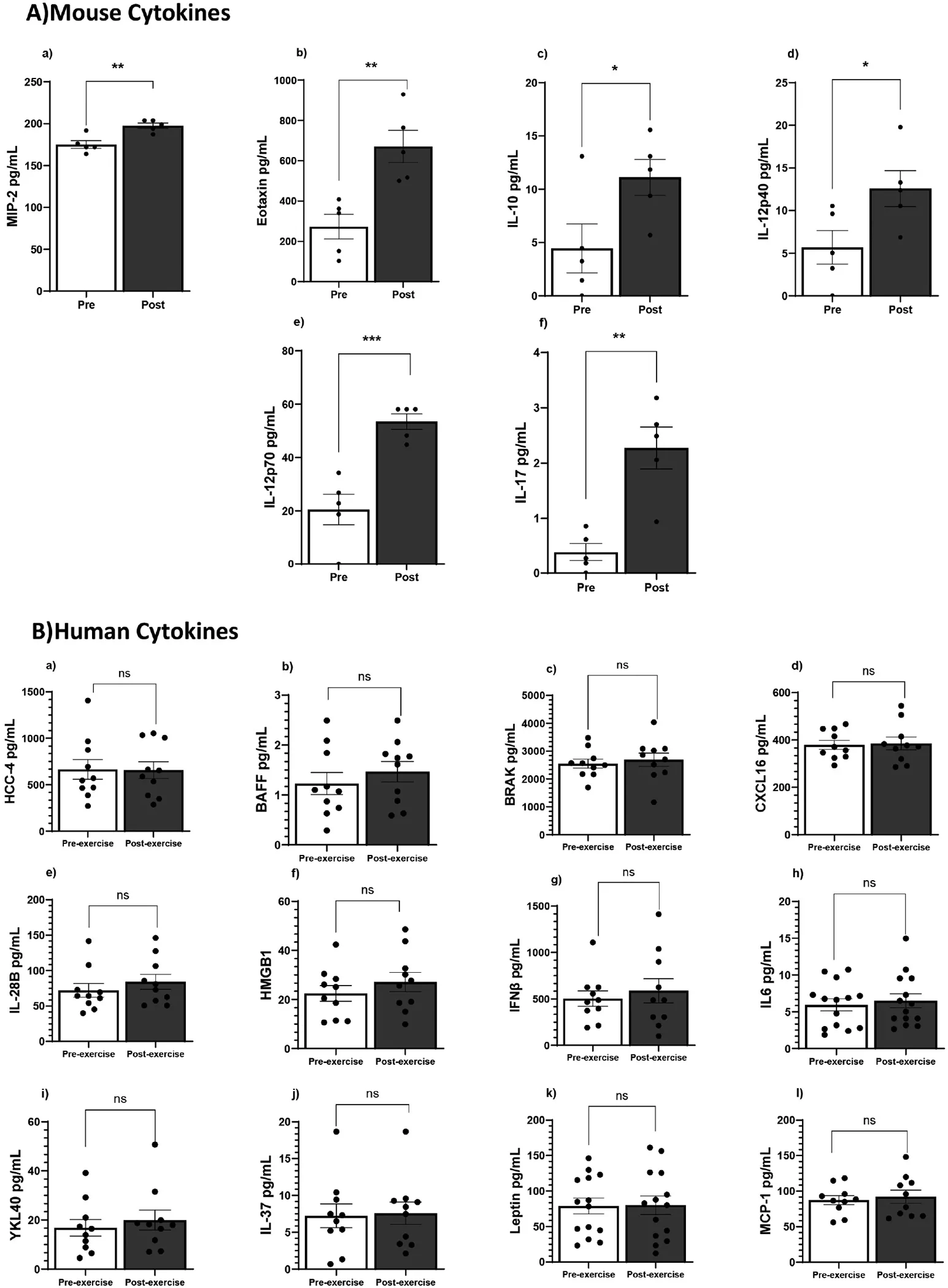
Plasma multiplex quantification analysis evidence exercise conditioning increases cytokines, chemokines, and growth factors, primarily in mice. Change of cytokine levels in humans after 16 weeks of exercise and 8 weeks of treadmill training in mice. Change of cytokine levels in humans after 16 weeks of exercise and 8 weeks of treadmill training in mice. (A) includes cytokines for mice: (a) MIP-2, (b) Eotaxin, (c) IL-10, (d) IL-12p40, (e) IL-12p70, (f) IL-17. (B) includes cytokines for humans: (a) HCC-4, (b) BAFF, (c) BRAK, (d) CXCL16, (e) IL-28B, (f) *HMGB1*, (g) IFN*β*, (h) IL-6, (i) YKL40, (j) IL-37, (k) Leptin, (l) MCP-1. All data represented mean ± SEM. All cytokine levels in post-exercise compared to pre-exercise of humans (n = 10) and mice (n = 5) were used for independent student *t*-test analysis. Statistical significance was set at a *p* value: ns, non-significant, *: <0.05, **: 0.001–0.01, ***: 0.0001. IL, Interleukin; HCC-4, C-C motif chemokine ligand 16; BAFF, B-cell activating factor; BRAK, Breast and kidney-expressed chemokine; CXCL16, C-X-C motif chemokine ligand 16; *HMGB1*, High Mobility Group Box 1; IFN*β*, Interferon beta; MCP-1, Monocyte chemoattractant protein-1; SEM, standard error of the mean; MIP-2, Macrophage inflammatory protein-2.

**Fig. 2.**
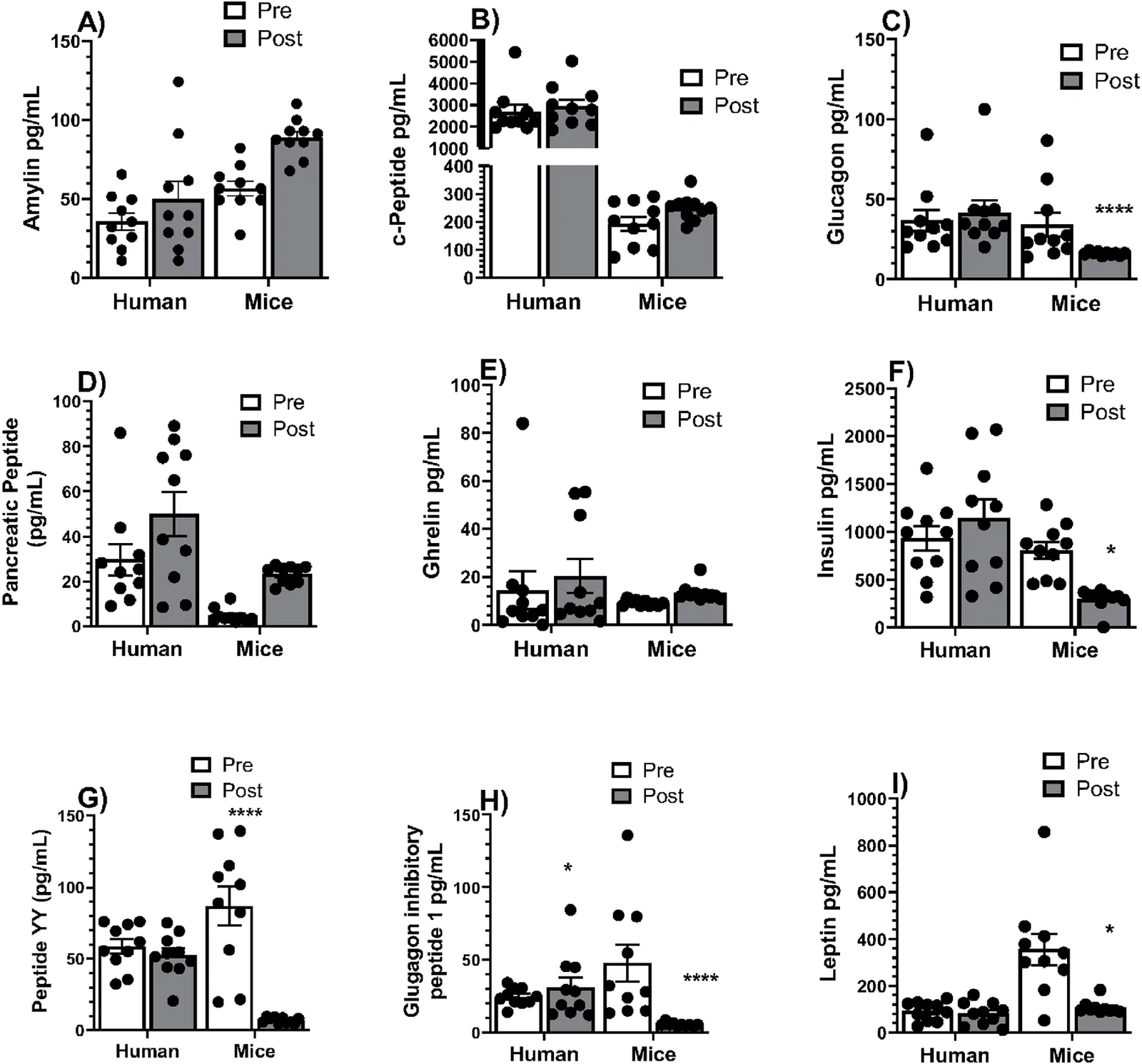
Plasma multiplex analysis evidence heterogeneity in hormonal benefits post-exercise between human and mouse exercise conditioning. Changes in hormonal levels in humans after 16 weeks of exercise and 8 weeks of treadmill exercise training in mice. Matched comparisons are shown pre- and post-exercise in both humans and mice for: (A) Amylin, (B) c-peptide, (C) Glucagon, (D) Pancreatic peptide, (E) Ghrelin, (F) Insulin, (G) Peptide YY, (H) Glucagon-like peptide-1 and (I) Leptin. All data represented mean ± SEM. All hormone levels in post-exercise compared to pre-exercise of humans (n = 10) and mice (n = 5) were used for independent student *t*-test analysis. Statistical significance was set at a *p* value: *: <0.05, ****: <0.0001.

**Fig. 3.**
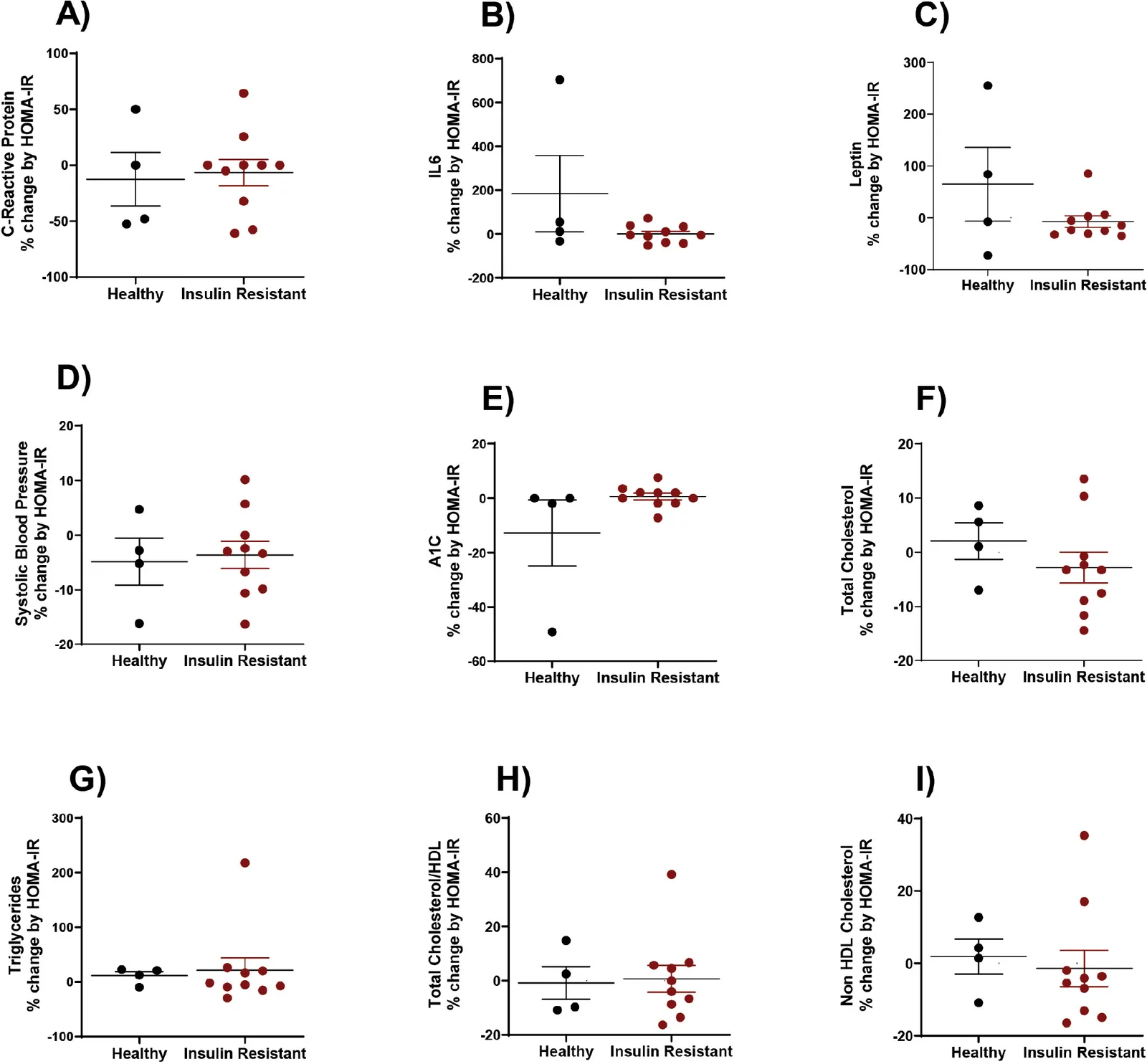
BW-HIIT enhances post exercise cardiometabolic benefits predominantly in insulin resistant individuals with high HOMA-IR. Standard of care and plasma cardiometabolic assessment evidenced the percentage of change sorted by HOMA-IR. Non-insulin resistant (<2 HOMA-IR) vs. insulin resistant (>2 HOMA-IR) individuals are presented for the following parameters: (A) C-Reactive Protein, (B) IL-6, (C) Leptin, (D) Systolic blood pressure, (E) HbA1c, (F) Total cholesterol, (G) Triglycerides, (H) Total cholesterol/HDL, (I) Non-HDL cholesterol. All data represented mean ± SEM. There is no statistical difference. All cytokine levels in insulin resistant (n = 10) compared to non-insulin resistant (n = 4) were used for independent student *t*-test analysis. BW-HIIT, Bodyweight high-intensity Interval Training; HOMA-IR, Homeostatic Model Assessment for Insulin Resistance; HDL, high-density lipoprotein.

**Fig. 4.**
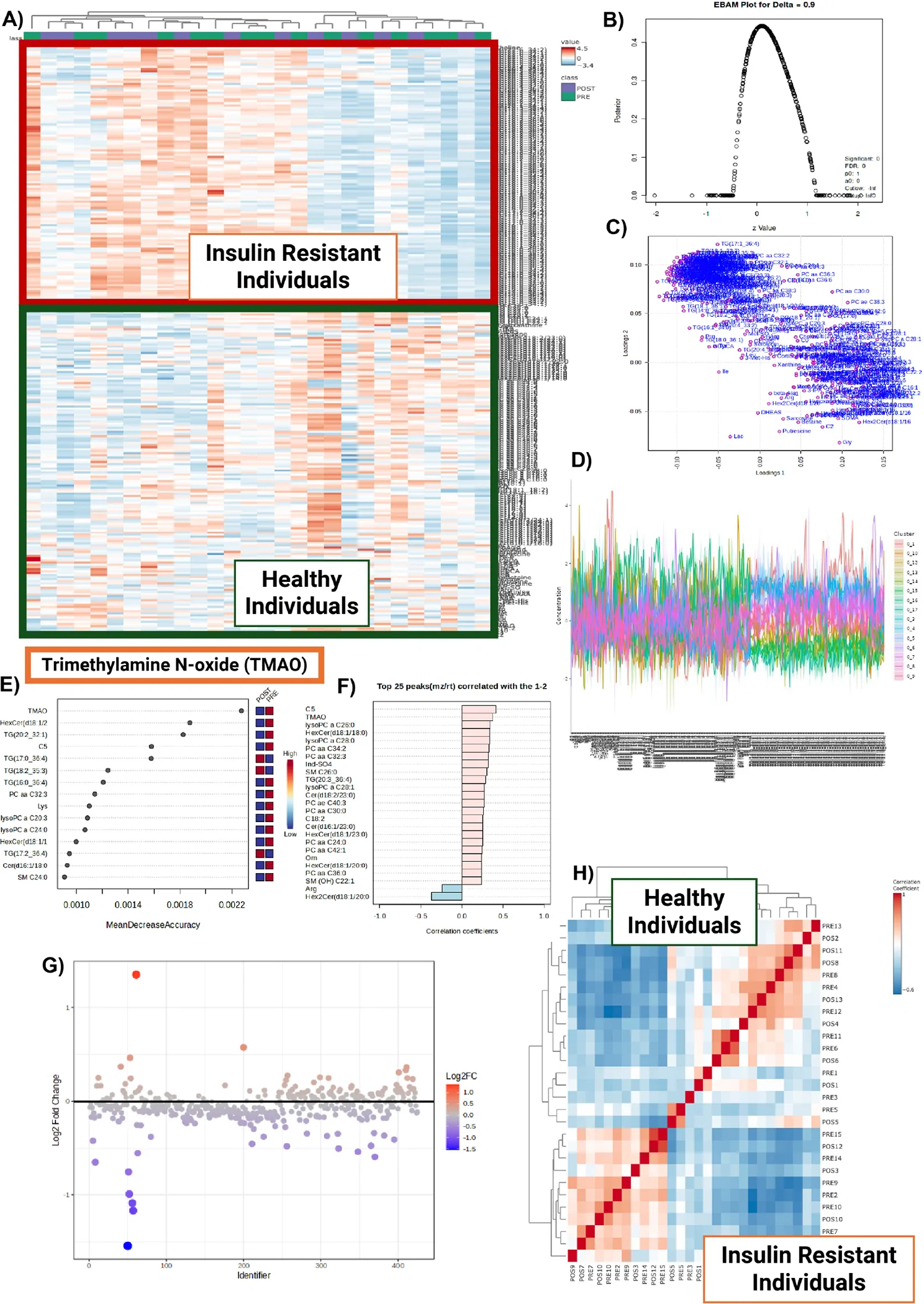
Plasma lipidomics identified overriding clustered differences post-exercise in insulin resistant individuals. (A) Heat map of principal changes between pre- vs. post-exercise changes between non-insulin resistant and insulin resistant individuals, (B) EBAM plot for delta data presentation, (C) PCA loading plot, (D) SOM plot for variability in sample data, (E) SPLS plot, (F) OPLS plot, (G) FC plot from post vs. pre-exercise score plot, (H) linear correlation heat map between non-insulin resistant vs. insulin resistant individuals after exercise. All data represented mean ± SEM. N of 4 for non-insulin resistant and 10 for insulin resistant patient groups. EBAM, empirical bayesian analysis of microarray; PCA, principal component analysis; SOM, self-organizing maps; SPLS, supervised partial least squares; OPLS, orthogonal partial least squares; FC, fold change.

**Fig. 5.**
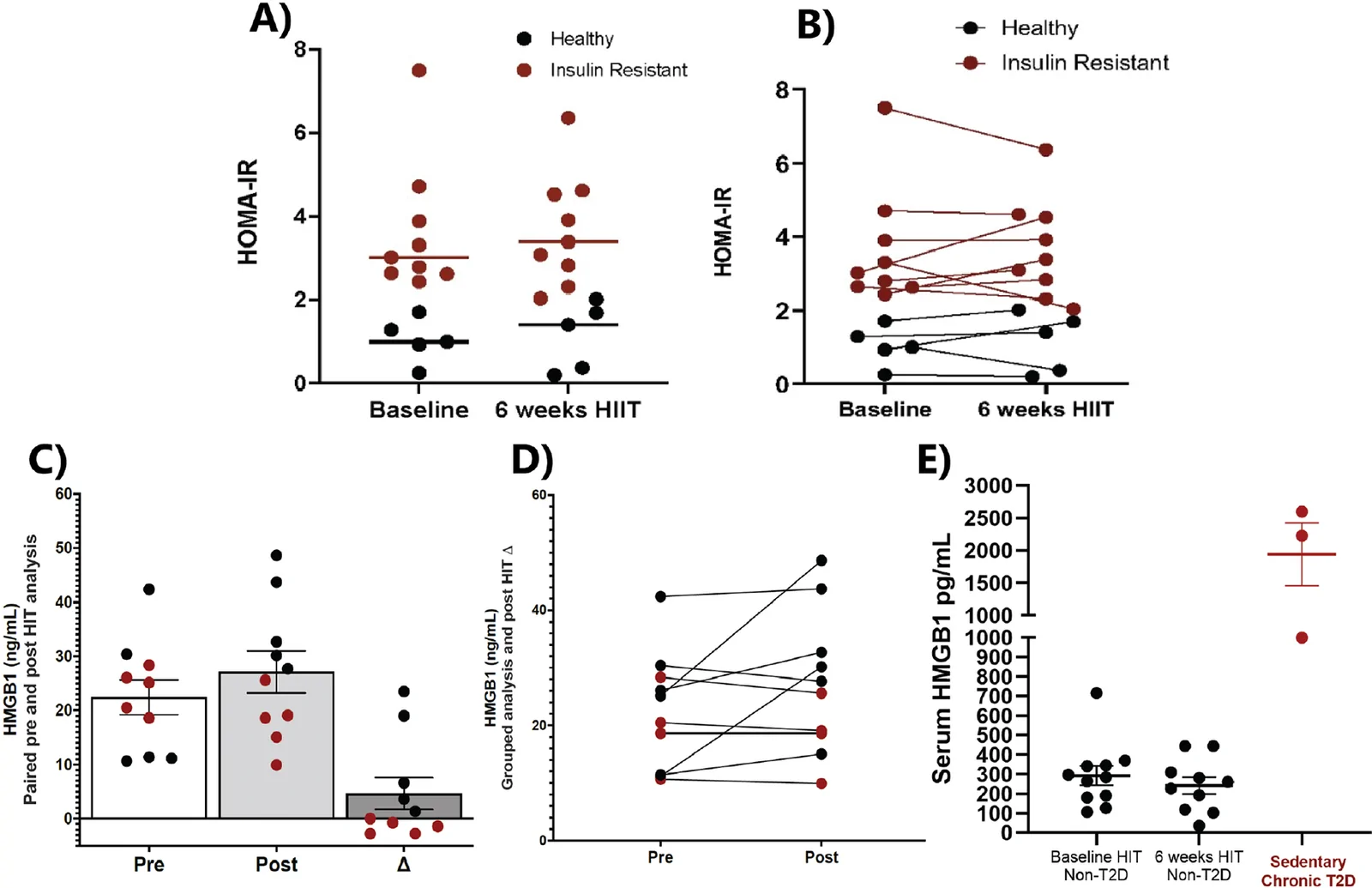
*HMGB1* and HOMA-IR levels share associated and decreases post-exercise in insulin resistant individuals. (A) HOMA-IR clustered-segmented side by side comparison (n = 5, non-insulin resistant; n = 9, insulin resistant), (B) HOMA-IR paired comparison (n = 5, non-insulin resistant; n = 9, insulin resistant), (C) Delta change in HMBG1 levels in pre and post-exercise (n of 10 for pre and post exercise), (D) HMBG1 levels in pre and post-exercise individually paired data presented (n of 10 for pre and post exercise), (E) Serum HMBG1 levels at baseline compared to 6 weeks after BW-HIIT of non-T2D compared to sedentary chronic T2D (n of 11 baseline BW-HIIT; n of 10 for 16 weeks BW-HIIT and n of 3 for Sedentary chronic T2D). All data represented mean ± SEM. Statistical analysis was performed independently using two-tailed student *t*-test analysis. T2D, Type 2 Diabetes.

## Data Availability

The data that support the findings is available in the MENDELEY DATA repository: Mota Alvidrez, Roberto (2024), “High-Intensity Interval Training Decreases Circulating HMGB1 in Insulin Resistant Individuals; Plasma Lipidomics Identifies Associated Cardiometabolic Benefits”, Mendeley Data. Other datasets used and/or analyzed during the current study are available from the corresponding author on reasonable request.
