# Supplementary material for: High-Intensity Interval Training Decreases Circulating HMGB1 in Individuals with Insulin Resistance: Plasma Lipidomics Correlate with Associated Cardiometabolic Benefits
Source: Front Biosci (Landmark Ed). Author manuscript; Available in PMC 2026 Oct 16. (PMC13674640; doi:10.31083/FBL31396)
Supplement: Supplementary file 1 [file NIHMS2209545-supplement-Supplemental_material.docx]

Supplemental Figures

**Supplementary Fig. 1. Unmatched plasma levels of hormones in pre and post exercise samples from mice.** Analysis shows comparison between pre and post treadmill at levels of Secretin (A) and Resistin (B). All data represented mean ± SEM. All cytokine levels in post-exercise (n = 5) compared to pre-exercise (n = 5) were used for independent student *t*-test analysis.

**Supplementary Fig. 2. Comparison of HMBG1 levels in distinct mouse models of Insulin Resistance and T2D. (**A) HMBG1 levels in sedentary (n = 3) vs. exercise mice (n = 5); (B) HMBG1 levels in post-exercise non-T2D (n = 6), insulin resistance (n = 3), and T2D mice (n = 3); and (C) HMBG1 levels in HMBG1 Flox mice fed with HFD and HMBG1 Flox mice fed with HFD and STZ. All data represented mean ± SEM. Independent student *t*-test analysis performed between groups. Statistical significance was set at a *p* value: ***: 0.0001.
